# A Practical Treatise on Headache, Etc.

**Published:** 1891-03

**Authors:** 


A Practical Treatise on Headache, Neuralgia, Sleep and its Derangements, and Spinal Irritation. By J. Leonard Corning, M. A., M. D., Consultant in Nervous Diseases at St. Francis Hospital ; Fellow of the New York Academy of Medicine ; Member of the New York Neurological Society, etc. Second edition with an appendix— Eye-Strain, a Cause of Headache. By David Webster, M. D., Professor of Ophthalmology in the New York Polyclinic ; Surgeon to the Manhattan Eye and Ear Hospital, etc., etc. In one octavo volume, nearly 300 pages. Price, $2.75. Uniform in style with Medical Classics. Special rate for the set. New York : E. B. Treat, publisher, 5 Cooper Union. 1890.

The second edition of this popular work is now given to the profession, with some important additions, especially the chapter on Eye-Strain, by Dr. Webster, of New York. The chapters on Headache—some sixty pages,—treat more of idealism in these cases than realism, and leaves the reader after all to make his own diagnosis and treatment. The subject matter is condensed too much to give very positive information, such as one would seek in treatises and monographs on special subjects. The chapters on Neuralgia are more complete and redundant in therapeutical measures. The author's ingenious appliances are carefully and fully described, giving the idea that there is still some room for originality in the discussion of this malady. The chapters on Insomnia and Spinal

Irritation, although we question this right of place in a "Treatise on Headache and Neuralgia," are well written and deserve more prominence than closing chapters in a book of this description.

The chapter on Eye-Strain will be read with much interest, in view of the recent conflict between Dr. Stevens and the New York Neurological Society.

The typographical work is uniform with the other volumes of medical classics, and needs no description to the reader save that it is on the same high level. W. C. K.

